# Serum oestrogen receptor *α* and *β* bioactivity are independently associated with breast cancer: a proof of principle study

**DOI:** 10.1038/sj.bjc.6605106

**Published:** 2009-06-02

**Authors:** M Widschwendter, H Lichtenberg-Frate, G Hasenbrink, S Schwarzer, A Dawnay, A Lam, U Menon, S Apostolidou, E Raum, C Stegmaier, I J Jacobs, H Brenner

**Affiliations:** 1 The Institute for Women's Health, University College London, London W1T 7DN, UK; 2 Department of Molecular Bioenergetics University Bonn, Bonn 53115, Germany; 3 Department of Clinical Biochemistry, University College London Hospitals NHS Foundation Trust, London W1T 4EU, UK; 4 The Division for Clinical Epidemiology and Ageing Research, German Cancer Research Centre, Heidelberg 69115, Germany; 5 The Saarland Cancer Registry, Saarbrücken 66024, Germany

**Keywords:** serum bioactivity, oestrogen receptor, breast cancer, risk

## Abstract

**Background:**

Oestrogens play a crucial role in breast carcinogenesis. Earlier studies have analysed the serum levels of endogenous hormones measured by conventional assays. In this study, we analysed the capacity of serum from breast cancer cases and controls to transactivate the oestrogen receptor *α* (ER-*α*) and *β* (ER-*β*).

**Methods:**

We used a receptor oestrogen-responsive element (ERE) – the green fluorescent protein (GFP)-reporter test system in *Saccharomyces cerevisiae*. Oestrogen receptor-*α* or ER-*β* bioactivity was determined in serum from 182 randomly chosen postmenopausal women with breast cancer and from 188 age-matched controls.

**Results:**

High serum ER-*α* and ER-*β* bioactivity were independently associated with the presence of breast cancer. Women whose levels of serum ER-*α* and ER-*β* bioactivity were in the highest quintile among controls had a 7.57-(95% confidence interval (CI): 2.46–23.32; *P*=0.0004) and a 10.14 (95% CI: 3.19–32.23; *P*<0.0001)-fold risk for general and oestrogen receptor-positive breast cancer, respectively.

**Conclusion:**

The use of serum ER-*α* and ER-*β* bioactivity assays as clinical tools in the management of breast cancer warrants further research. Future studies will dictate whether surrogate markers of ER-*α* and ER-*β* bioactivity will provide a means to monitor the efficacy of anti-endocrine, adjuvant and chemopreventive strategies.

Oestrogens are known to play a crucial role in breast carcinogenesis. High serum oestrogen levels (Toniolo *et al*, 1995; Thomas *et al*, 1997; Hankinson *et al*, 1998; Cauley *et al*, 1999; Key *et al*, 2002; Missmer *et al*, 2004) and surrogates for long-term high oestrogen exposure, such as bone (Zhang *et al*, 1997) density, are known to be associated with an increased risk for breast cancer. However, exposure to anti-oestrogens has been shown to decrease the risk for breast cancer and recurrent disease (Baum *et al*, 2002; Cuzick *et al*, 2003). A retrospective analysis of worldwide data in postmenopausal women showed that elevated levels of endogenous oestrogens further from the time of diagnosis seem to indicate a greater risk for breast cancer than equivalent levels immediately before diagnosis (Key *et al*, 2002).

Standard assays of steroid hormones, including oestrogens, are based on various immunological detection methods. Although these assays are widely used in routine clinical diagnosis and research, a major limitation is that they do not reflect the level of hormonal bioactivity in the sample, but rather the combined immunoreactivity of compounds structurally related to the immunogen.

Oestrogens exert their action by binding to intracellular oestrogen receptor (ER) proteins that, on dimerisation, bind to specific DNA sequences (oestrogen-responsive elements (EREs)) in the regulatory regions of target gene promoters. The degree of transcriptional stimulation in a particular cell type depends on the pattern and concentration of the activated ER proteins and several complex regulating factors. In addition to oestrogen metabolites, environmental contaminants such as polycyclic aromatic hydrocarbons, phthalates, pesticides and certain plant constituents, termed phytoestrogens, bind and activate ER proteins (Cvoro *et al*, 2007; Heldring *et al*, 2007). Both oestrogen receptor *α* (ER-*α*) and *β* (ER-*β*) have been shown to play independent and fundamental roles in breast carcinogenesis (Yager and Davidson, 2006).

All earlier studies have only explored levels of endogenous hormones with regard to breast cancer risk. Over the last decade, several groups have developed bioactivity assays for steroid hormones mainly to detect very-low hormone levels in children (Klein *et al*, 1994, 1998; Paris *et al*, 2002; Roy *et al*, 2006). Recently, we have described a receptor ERE-green fluorescent protein (GFP)-reporter test system in *Saccharomyces cerevisiae*, which measures activation of ER-*α* and ER-*β* independently. This transactivation assay involves ER-*α* or ER-*β* receptors coupled to the ERE-GFP-reporter in a genetically modified yeast strain, devoid of three endogenous xenobiotic transporters (*PDR5*, *SNQ2* and *YOR1*) (Figure 1) (Sievernich *et al*, 2004; Hasenbrink *et al*, 2006).

In this paper, we analyse for the first time, oestrogen bioactivity in serum from breast cancer cases and controls in addition to endogenous hormone levels measured by a conventional immunoassay.

## Materials and methods

### Patients

All serum samples were collected as part of the population-based study ESTHER, carried out in the state of Saarland, Germany. In the hospital arm of this study, 1981 cancer patients aged 50–75, including 380 women with breast cancer, were recruited after diagnosis and at the first hospitalisation for initial cancer treatment. In the community arm, 9953 women and men aged 50–75 were recruited during routine health examinations by their general practitioners. Recruitment and baseline examinations were carried out between 2000–2003. In both study arms, detailed information of medical history, including family history, socio-demographics, lifestyle factors and current health status, was obtained using a standardised questionnaire. In addition, serum and whole blood samples were collected. Serum samples were sent, after centrifugation, to the study centre by regular mail, where they were aliquoted and frozen at −80°C until analysis. Procedures for the collection, processing and storing of blood samples were identical for both cases and controls. The written informed consent was obtained from all subjects for use of the data and samples in future ethically approved research. The protocol was approved by the Ethics Committee of the Medical Faculty of the University of Heidelberg, Germany. For the current study, we analysed serum samples from 182 randomly chosen postmenopausal women with breast cancer (mean age 62.1 years) and from 188 age-matched controls (mean age 62.5 years).

### Serum oestradiol assay

Serum oestradiol was analysed on the Roche E170 analyser (Roche Diagnostics GmbH, Mannheim, Germany) using a competitive chemiluminescence immunoassay after release from binding proteins by mesterolone. The assay uses 35 *μ*l of sample and has a quoted detection limit of 5 pg ml^−1^ with a functional sensitivity (inter-assay coefficient of variation (CV)<20%) of 12 pg ml^−1^, intra-assay CV of 3.3% at 35.5 pg ml^−1^ and inter-assay CV of 6.2% at 22.9 pg ml^−1^. The samples were analysed blind in batches over 4 days using a single lot number of reagent and calibrator.

### ER-*α* and ER-*β* serum bioactivity assay

Serum oestradiol bioactivity was assessed using the transactivation assay consisting of genetically modified yeast expressing the human ER-*α* or ER-*β* receptor, as described earlier (Sievernich *et al*, 2004; Hasenbrink *et al*, 2006). The analysis was performed blinded, and cases and controls were randomly mixed and blinded to the group at the University of Bonn, where the assays have been performed. In brief, for quantitative assessment of growth phenotypes and fluorescence development, logarithmic growing cells were exposed to 20 *μ*l of serum aliquots. Tests were carried out with two replicates at a time on two different days (thus four readings in total) in transparent 96-well microtitre plates using a microplate reader. Each experiment comprised of 0–10 000 pg ml^−1^ oestradiol concentration ranges (in 20% charcoal-stripped serum) as reference values and positive controls. Tests were considered as valid when the turbidity of the control cultures increased at least five-fold during the incubation period of 16.5 h. End-point fluorescence values were corrected for blanks and normalised for cell number. Serum ER-*α* and ER-*β* bioactivity were calculated from the oestradiol reference curve and expressed as equivalent oestrogen activity accordant to oestradiol in pg ml^−1^. Dose-response curves of the oestradiol reference values were fitted as described earlier using the Hill equation fit and the R function *nls* (The R Foundation for Statistical Computing, http://www.r-project.org/) (Sievernich *et al*, 2004; Hasenbrink *et al*, 2006). No temporal effects could be detected. By the nature of the assay, minor daily performance differences may occur, but were accounted for by including a daily reference curve, comprising 10 different estradiol concentrations. Order effects were not detected since, as mentioned above, all samples were randomised before numerical coding.

### Statistics

Breast cancer cases were described with respect to major tumour characteristics and ER status (ER-positive and ER-negative) by immunohistochemistry of the original tumour. Cases and controls were described with respect to established breast cancer risk factors. Mean and median levels of oestradiol, serum ER-*α* and ER-*β* bioactivity were calculated for ER-positive, ER-negative, all breast cancer samples and for controls. Differences in the means between groups were tested for statistical significance using the Kruskal–Wallis test. Correlations between oestradiol, serum ER-*α* and ER-*β* bioactivity among cases and controls were assessed by the Spearman's rank correlation coefficient. The associations between oestradiol, serum ER-*α* and ER-*β* bioactivity levels, and the risk of ER-positive, ER-negative and total breast cancer risk, were determined after controlling for age by multiple logistic regression. Subjects were classified according to quintiles of the respective marker among controls. Finally, serum ER-*α* and ER-*β* bioactivity levels were jointly entered and controlled for in the regression models to estimate their independent and combined associations with breast cancer risk.

## Results

A total of 14 women with breast cancer and 63 controls were excluded as they were using hormone replacement therapy at the time of blood collection. The epidemiological risk factor profile for the remaining 168 cases and 125 controls is shown in Supplementary Table 1. Cases were significantly more likely to have first-degree relatives with breast cancer (odds ratio (OR) 3.16; 95% confidence interval (CI): 1.09–9.21). Tumour characteristics of the cases were similar to a typical breast cancer cohort (Supplementary Table 2).

No difference was observed in serum oestradiol levels between cases and controls (Supplementary Table 3). Serum ER-*β* bioactivity was only detectable in 36 and 48.8% of controls and cases, respectively. However, both serum ER-*α* and ER-*β* bioactivity showed significant differences between all cases and controls (Table 1). A difference for both ER-*α* and ER-*β* serum bioactivity was shown between ER-positive breast cancer cases and controls. In addition, there was also a clear trend, although not significant, towards higher serum ER-*β* bioactivity in ER-negative breast cancer cases compared with controls (Table 1).

Using all samples, no significant correlations among oestradiol, ER-*α* and ER-*β* serum bioactivity were observed. Using only samples with oestradiol above the lower threshold of the test (>12 pg ml^−1^) showed a positive correlation between serum ER-*α* bioactivity and oestradiol (Table 2). A borderline association between ER-*α* and ER-*β* serum bioactivity was only observed after samples with undetectable ER-*β* serum bioactivity have been excluded.

The values for oestradiol, ER-*α* and ER-*β* serum bioactivity were classified according to quintiles among controls. No association with serum oestradiol could be observed (data not shown), but both ER-*α* and ER-*β* serum bioactivity were significantly increased in breast cancer samples. Women with ⩾42.1 pg ml^−1^ serum ER-*α* bioactivity and >53 pg ml^−1^ ER-*β* serum bioactivity had a 2.47-(95% CI: 1.17–5.20) and 2.34 (95% CI: 1.33–4.13)-fold risk for breast cancer and 2.70-(95% CI: 1.23–5.90) and 2.31 (95% CI: 1.27–4.22)-fold risk for ER-positive breast cancer, respectively. A trend towards higher risk of ER-negative breast cancer with high levels of ER-*β* serum bioactivity was observed (OR 2.27 (95% CI: 1.00–5.15)) (Table 3A and 3B).

In order to test whether the ER-*α* and ER-*β* serum bioactivity are independently associated with breast cancer, a logistic regression analysis was performed adjusting for the bioactivity of the other receptor, and for age. Both ER-*α* and ER-*β* serum bioactivity were independently associated with general and ER-positive breast cancer. Oestrogen receptor-*β* serum bioactivity, after adjustment for ER-*α* serum bioactivity and age, was significantly associated with general (*P*=0.008) and with ER-positive (*P*=0.01) breast cancer. A borderline significant association (*P*=0.07) between ER-*β* serum bioactivity and ER-negative breast cancer emerged after adjustment for ER-*α* serum bioactivity (Table 4).

To test whether a combined approach would increase the predictive power, the groups defined by first–fourth and fifth quintile were analysed. Women whose ER-*α* and ER-*β* serum bioactivity were in the fifth quintile of controls had a 7.57-(95% CI: 2.46–23.32) and a 10.14 (95% CI: 3.19–32.23)-fold risk for general and ER-positive breast cancer, respectively. Women with first–fourth quintile ER-*α* serum bioactivity and fifth quintile ER-*β* serum bioactivity had a 2.36-(0.97–5.69) fold risk for ER-negative breast cancer (Table 5). The observed patterns were independent of the defined cutoff, although the strength of associations increased with the increasing cut-points: Women whose ER-*α* and ER-*β* serum bioactivity were in the top tertile, quartile or >90 percentile had a 4.47-(95% CI: 2.06–9.70; *P*=0.0002), 5.52-(95% CI: 2.34–13.01; *P*<0.0001) or 12.76 (95% CI: 1.60–101.97; *P*=0.16)-fold risk for general and a 5.30-(95% CI: 2.34–11.99; *P*<0.0001), 7.16-(95% CI: 2.92–17.57; *P*<0.0001) or 19.47 (95% CI: 2.38–159.03; *P*<0.0001)-fold risk for ER-positive breast cancer, respectively, compared with women with ER-*α* and ER-*β* serum bioactivity below the respective cutoff points.

## Discussion

In this paper, we describe for the first time a yeast-based serum bioactivity assay to predict the presence of breast cancer. To date, there have been no reports showing such a significant association between the high serum hormone levels and the presence of any endocrine-related cancer.

In postmenopausal women, both ER-*α* and ER-*β* serum bioactivity were significantly different between patients with breast cancer and age-matched controls, whereas there was no difference in serum oestradiol levels between the two groups. The oestradiol findings are in keeping with a recent retrospective analysis of data from prospective studies, in which neither total (relative risk 1.21, 95% CI: 0.99–1.47) nor free serum oestradiol (relative risk 1.18, 95% CI: 0.98–1.43) was an increased risk marker for women who were diagnosed with breast cancer shortly after the blood collection (Key *et al*, 2002). Our results show that the serum bioactivity assay, which estimates the total functional oestrogenic activity, is better than conventional hormonal assays for estimating breast cancer risk. The assay is able to quantify the effect of all circulating active oestrogen metabolites, as well as other substances that can activate the oestrogen receptors and induce gene transcription.

In the retrospective analysis referred to above, analysis of the majority of hormones in postmenopausal women shows a somewhat greater cancer risk in patients who were diagnosed 2 or more years after blood collection than in patients who were diagnosed within 2 years of blood collection (Key *et al*, 2002). This suggests that these functional assays may have more predictive power when analysing samples preceding diagnosis and, as a consequence, could provide an excellent means of screening for breast cancer. A study, supportive of this view, is currently underway using samples from women who developed cancer in the UK Collaborative Trial of Ovarian Cancer Screening.

In ER-positive breast cancer, the combination of high-ER-*α* and high-ER-*β* serum bioactivity were associated with a 10.14-fold risk of breast cancer. This is far higher than the 2–2.5-fold risk reported with elevated serum oestrogenic activity measured using hormonal assays in similar case–control studies. The bioactivity assay, unlike conventional hormonal measurements, is able to act as a surrogate biomarker for the potential of all factors in the serum to transactivate the two different ERs, namely ER-*α* and ER-*β*. Activation of ER-*α* by means of oestradiol and inhibition of ER-*α* by means of tamoxifen, or indirectly by means of aromatase inhibitors, increases and decreases ER-positive breast cancer risk, respectively (Cuzick *et al*, 2003; Yager and Davidson, 2006).This is consistent with our finding that ER-*α* serum bioactivity predicts ER-positive, but not ER-negative breast cancer.

Entirely unexpectedly, high levels of ER-*β* serum bioactivity, after adjustment for ER-*α* serum bioactivity and age, were found to be associated with increased risk for ER-positive and ER-negative breast cancer. Although it is generally assumed that the growth of ER-negative breast cancers is not influenced by oestrogens, there is an evidence to suggest that oophorectomy prevents the formation of both ER-positive and ER-negative breast cancers (Nissen-Meyer, 1964; Early Breast Cancer Trialists' Collaborative Group, 1992), indicating that even ER-negative breast cancers may depend on hormones for their formation. *BRCA1*-associated tumours, the vast majority of which are ER-negative, are also effectively prevented by removal of the ovaries (Rebbeck *et al*, 2002). Recent evidence suggested that oestrogens facilitate ER-negative breast carcinogenesis by modulating stromal components (Gupta *et al*, 2007). The main component of breast stroma, namely mammary fibroblasts, only expresses ER-*β* but lack expression of ER-*α* (Palmieri *et al*, 2004). A simple explanation for our finding could be that compounds in the serum-activating ER-*β* (as reflected by high-ER-*β* serum bioactivity) facilitate ER-negative breast carcinogenesis by activating breast fibroblasts.

Interestingly, there was only a weak correlation between oestradiol and ER-*α* serum bioactivity and only in women with oestradiol levels higher than the threshold for functional sensitivity (>12 pg ml^−1^). In addition, we could not find a correlation between oestradiol and ER-*β* serum bioactivity, although oestradiol is known to bind to both receptors. ER-*α* and ER-*β* serum bioactivity, independent of each other predicts the presence of breast cancer. Compared with the ‘best ligand’ oestradiol, the corresponding ER-*α* and ER-*β* serum bioactivity are at least 2–3 times higher (data not shown). This can be explained by the presence of a variety of additional oestrogenic compounds in the serum, which transactivate both ER proteins in different ways (i.e., 17-*α*-oestradiol, phytoestrogens and so on) (Sievernich *et al*, 2004; Hasenbrink *et al*, 2006). In addition, non-steroidal factors in the serum, which have an impact on the capacity to transactivate ER-*α* or ER-*β*, may also contribute to the higher ER-*α* and ER-*β* bioactivity compared with oestradiol levels in serum. It is known that steroid-independent pathways exist for activation of a steroid receptor through signalling cascades from membrane-regulatory molecules, such as cAMP, dopamine, growth factors, cytokines, and possibly other cellular regulators acting at the membrane (Aronica and Katzenellenbogen, 1993; Ignar-Trowbridge *et al*, 1993; O'Malley, 2005). Therefore, we speculate that the serum contains steroid-independent co-activators, which have an impact on breast carcinogenesis and are reflected by ER-*α* and ER-*β* serum bioactivity.

Two types of immunoassay can be used to measure total oestradiol: indirect assays use a pre-analytical extraction step with organic solvent to dissociate oestradiol from its binding proteins and remove potentially cross-reacting hydrophilic steroid conjugates, whereas direct assays employ an agent with greater affinity for the binding of proteins that then displace oestradiol. Indirect assays are typically more sensitive and accurate. Here, we used a direct automated immunoassay. This may be an additional explanation for the lack of correlation between oestradiol and ER-*α* or ER-*β* serum bioactivity.

In summary, serum bioactivity assays for both ER-*α* and ER-*β* predict ER-positive breast cancer at the time of diagnosis. In addition, the serum bioactivity of ER-*β* controlled for the activity of ER-*α* is tentatively associated with ER-negative breast cancer. The findings of this proof of principle study may have the potential to open up an entirely new window of opportunity to: (a) predict breast cancer and (b) allow monitoring of preventive and therapeutic hormonal therapies (tamoxifen or aromatase inhibitors) in breast cancer. These issues need to be addressed in future studies.

## Figures and Tables

**Figure 1:**
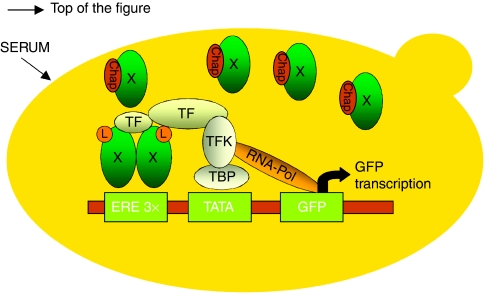
Principle of the oestrogen receptor bioactivity assay enabling investigation of receptor-mediated transcriptional activation of the green fluorescent protein optimised for expression in yeast (yEGFP3) under the control of oestrogen-responsive elements (EREs). X: oestrogen receptor-*α* (ER-*α*) or oestrogen receptor-*β* (ER-*β*), respectively; ERE 3 × : three-fold repeat of 5′-GGGTCACAGTGACCGCTAG-3′.

**Table 1:** Oestrogen receptor *α* (ER-*α*) and ER-*β* serum bioactivity in serum samples from 125 controls and from168 breast cancer cases

	**Group**			
		**Cases**		
**Characteristic**	**Controls**	**All**	**ER-negative**	**ER-positive**
*N*	125	168	42	126
				
*ER-α serum bioactivity (pg ml* ^*−1*^ *)*				
Range	0–111.8	0–298.3	6.7–131.8	0–298.3
Mean	30.5	50.7	33.9	56.3
Median	25.6	33.0	27.9	37.1
*P*-value^a^		*P*=0.002	*P*=0.18	*P*=0.001
				
*ER-β serum bioactivity (pg ml* ^*−1*^ *)*				
Range	0–315.7	0–844.6	0–583.7	0–844.6
Mean	37.4	93.5	77.9	98.7
Median	0.0	0.0	0.0	0.0
*P*-value^a^		*P*=0.004	*P*=0.08	*P*=0.005

a Kruskal–Wallis test for difference in mean value among case group and controls.

**Table 2:** Correlation coefficients (Spearman) between oestrogen receptor *α* (ER-*α*), ER-*β* serum bioactivity and serum oestradiol

**Sample**	**ER-*α* and ER-*β* serum bioactivity**	**ER-*α* serum bioactivity and oestradiol**	**ER-*β* serum bioactivity and oestradiol**
Total (*N*=293)	0.08	0.08	0.00
	*P*=0.15	*P*=0.18	*P*=0.98
ER-*β* serum bioactivity >0 (*N*=127)	0.18	0.09	0.03
	*P*=0.05	*P*=0.30	*P*=0.74
Oestradiol >12 (*N*=178)	0.11	0.18	−0.05
	*P*=0.16	*P*=0.02	*P*=0.52
ER-*β* serum bioactivity >0 and oestradiol >12 (*N*=79)	0.22	0.18	0.10
	*P*=0.05	*P*=0.11	*P*=0.40

**Table 3a:** Association of oestrogen receptor *α* (ER-*α*) serum bioactivity with risk of breast cancer

			**Cases**					
**ER-*α* serum bioactivity (pg ml^−1^)**		**Controls**	**All**		**ER-negative**		**ER-positive**	
**Quintile^a^**	**Range**	***N* (%)**	***N* (%)**	**OR (95% CI)^b^**	***N* (%)**	**OR (95% CI)^b^**	***N* (%)**	**OR (95% CI)^b^**
1st	0–13.8	24 (19.1)	24 (14.3)	1.00 (ref.)	4 (9.5)	1.00 (ref.)	20 (15.9)	1.00 (ref.)
2nd	13.9–21.6	26 (20.8)	25 (14.9)	1.00 (0.45–2.23)	7 (16.7)	1.53 (0.39–6.06)	18 (14.3)	0.92 (0.39–2.18)
3rd	21.7–29.5	25 (20.0)	23 (13.7)	0.97 (0.43–2.17)	12 (28.6)	3.22 (0.88–11.77)	11 (8.7)	0.53 (0.21–1.36)
4th	29.6–42.0	25 (20.0)	33 (19.6)	1.24 (0.57–2.71)	10 (23.8)	2.18 (0.59–8.11)	23 (18.3)	1.09 (0.47–2.52)
5th	⩾42.1	25 (20.0)	63 (37.5)	2.47 (1.17–5.20)	9 (21.4)	1.84 (0.49–6.89)	54 (42.9)	2.70 (1.23–5.90)
*P*-value^c^				*P*=0.008		*P*=0.36		*P*=0.004

a Quintiles according to distribution among controls.

b Adjusted for age.

c *P*-value for trend, adjusted for age.

**Table 3b:** Association of oestrogen receptor *β* (ER-*β*) serum bioactivity with risk of breast cancer

			**Cases**					
**ER-*β* serum bioactivity (pg ml^−1^)**		**Controls**	**All**		**ER-negative**		**ER-positive**	
**Quintile^a^**	**Range**	***N* (%)**	***N* (%)**	**OR (95% CI)^b^**	***N* (%)**	**OR (95% CI)^b^**	***N* (%)**	**OR (95% CI)^b^**
1st–3rd	0	80 (64.0)	86 (51.2)	1.00 (ref.)	22 (52.4)	1.00 (ref.)	64 (50.8)	1.00 (ref.)
4th	0.1–53	20 (16.0)	21 (12.5)	0.97 (0.48–1.96)	4 (9.5)	0.77 (0.23–2.57)	17 (13.5)	1.03 (0.49–2.17)
5th	>53	25 (20.0)	61 (36.3)	2.34 (1.33–4.13)	16 (38.1)	2.27 (1.00–5.15)	45 (35.7)	2.31 (1.27–4.22)
*P*-value^c^				*P*=0.005		*P*=0.07		*P*=0.009

a Quintiles according to distribution among controls.

b Adjusted for age.

c *P*-value for trend, adjusted for age.

**Table 4:** Association of oestrogen receptor *α* (ER-*α*) and ER-*β* serum bioactivity (controlled for each other) with risk of breast cancer; classification according to quintiles among controls

			**Cases**					
		**Controls**	**All**		**ER-negative**		**ER-positive**	
**Quintile^a^**	**Range (pg ml^−1^)**	***N* (%)**	***N* (%)**	**OR (95% CI)^b^**	***N* (%)**	**OR (95% CI)^b^**	***N* (%)**	**OR (95% CI)^b^**
*ER-α serum bioactivity*								
1st	0–13.8	24 (19.2)	24 (14.3)	1.00 (ref.)	4 (9.5)	1.00 (ref.)	20 (15.9)	1.00 (ref.)
2nd	13.9–21.6	26 (20.8)	25 (14.9)	0.99 (0.44–2.27)	7 (16.7)	1.52 (0.38–6.16)	18 (14.3)	0.91 (0.38–2.22)
3rd	21.7–29.5	25 (20.0)	23 (13.7)	0.82 (0.35–1.91)	12 (28.6)	2.80 (0.73–10.67)	11 (8.7)	0.44 (0.17–1.18)
4th	29.6–42.0	25 (20.0)	33 (19.6)	1.07 (0.47–2.43)	10 (23.8)	2.02 (0.53–7.78)	23 (18.3)	0.94 (0.39–2.28)
5th	⩾42.1	25 (20.0)	63 (37.5)	2.24 (1.04–4.81)	9 (21.4)	1.80 (0.47–6.90)	54 (42.9)	2.43 (1.09–5.44)
*P*-value^c^				*P*=0.01		*P*=0.34		*P*=0.007
								
*ER-β serum bioactivity*								
1st–3rd	0	80 (64.0)	86 (51.2)	1.00 (ref.)	22 (52.4)	1.00 (ref.)	64 (50.8)	1.00 (ref.)
4th	0.1–53	20 (16.0)	21 (12.5)	0.94 (0.45–1.96)	4 (9.5)	0.92 (0.27–3.14)	17 (13.5)	0.93 (0.42–2.06)
5th	>53	25 (20.0)	61 (36.3)	2.32 (1.04–4.81)	16 (38.1)	2.12 (0.92–4.87)	45 (35.7)	2.36 (1.26–4.43)
*P*-value^c^				*P*=0.008		*P*=0.07		*P*=0.01

a Quintiles according to distribution among controls.

b Adjusted for age and other marker.

c *P*-value for trend, adjusted for age and other marker.

**Table 5:** Joint association of high oestrogen receptor *α* (ER-*α*) serum bioactivity (5th quintile) and high ER-*β* serum bioactivity (5th quintile) with risk of breast cancer

**Quintile^a^**			**Cases**					
**Range**			**Controls**	**All**		**ER-negative**		**ER-positive**
**ER-*α* serum bioactivity**	**ER-*β* serum bioactivity**	***N* (%)**	***N* (%)**	**OR (95% CI)^b^**	***N* (%)**	**OR (95% CI)^b^**	***N* (%)**	**OR (95% CI)^b^**
1st–4th	1st–4th	79 (63.2%)	69 (41.1%)	1.00 (ref.)	20 (47.6%)	1.00 (ref.)	49 (38.9%)	1.00 (ref.)
0–42 pg ml^−1^	0–53 pg ml^−1^							
5th	1st–4th	21 (16.8%)	38 (22.6%)	1.97 (1.05–3.72)	6 (14.3%)	0.99 (0.35–2.81)	32 (25.4%)	2.41 (1.23–4.72)
>42 pg ml^−1^	0–53 pg ml^−1^			*P*=0.04		*P*=0.98		*P*=0.01
1st–4th	5th	21 (16.8%)	36 (21.4%)	1.98 (1.05–3.75)	13 (31.0%)	2.36 (0.97–5.69)	23 (18.3%)	1.77 (0.88–3.58)
0–42 pg ml^−1^	>53 pg ml^−1^			*P*=0.03		*P*=0.06		*P*=0.11
5th	5th	4 (3.2%)	25 (14.9%)	7.57 (2.46–23.32)	3 (7.1%)	2.36 (0.47–11.79)	22 (17.5%)	10.14 (3.19–32.23)
>42 pg ml^−1^	>53 pg ml^−1^			*P*=0.0004		*P*=0.30		*P*<0.0001
*P*-value for trend^c^				*P*<0.0001		*P*=0.14		*P*<0.0001

a Quintiles according to distribution among controls.

b Adjusted for age.

c In an analysis combining categories 2 and 3, adjusted for age.
