# Supplementary material for: Serum oestrogen receptor α and β bioactivity are independently associated with breast cancer: a proof of principle study
Source: Br J Cancer. 2009 Jun 2;101(1):160–5. doi: 10.1038/sj.bjc.6605106 (PMC2713696; doi:10.1038/sj.bjc.6605106)
Supplement: Supplementary Tables 1–3 [file 6605106x1.doc]

**SUPPLEMENTARY TABLES**

**Supplementary Table 1.** Traditional risk factors in cases and controls

*adjusted for other variables in table; numbers of cases and controls do not always add up to totals due to missing values in some participants.

**Supplementary Table 2.** Tumour characteristics

**Supplementary Table 3.** Estradiol concentration in serum samples from 125 controls and 168 breast cancer cases

| Characteristic | Group | | | |
| --- | --- | --- | --- | --- |
|  | Controls | Cases | | |
|  |  | All | Estrogen receptor- negative | Estrogen receptor-positive |
| N | 125 | 168 | 42 | 126 |
| Estradiol [pg/ml] |  |  |  |  |
| Range | <12-237.1 | <12-156.5 | <12-156.5 | <12-83.8 |
| Mean | 20.3 | 17.7 | 18.4 | 17.5 |
| Median | 12.9 | 13.9 | 13.5 | 14 |
| *P*-value^*^ |  | *P*=0.66 | *P*1.00 | *P=*0.58 |

^*^ Kruskal-Wallis test for difference in mean value among case group and controls. In case estradiol concentration was <12 pg/ml, 12 pg/ml has been used to calculate the mean
